# From immunoinformatics insights to assay development: establishment of a peptide-based indirect elisa for cynomolgus tuberculosis detection

**DOI:** 10.3389/fcimb.2026.1799877

**Published:** 2026-06-19

**Authors:** Dengling Huang, Mingjie Chen, Qiuyu Wu, Pengwei Liu, Leting Yang, Weixiang Huang, Kunhui Mo, Xiao Wei, Ying Tian, Jianhua Sun, Likun Gong

**Affiliations:** 1 Nanjing University of Chinese Medicine, Nanjing, China; 2 Zhongshan Institute for Drug Discovery, Shanghai Institute of Materia Medica, Chinese Academy of Sciences, Zhongshan, China; 3 School of Pharmaceutical Sciences, Southern Medical University, Guangzhou, China; 4 Guangxi Zhuang Autonomous Region Terrestrial Wildlife Medical-aid and Monitoring Epidemic Diseases Research Center, Nanning, China; 5 State Key Laboratory of Drug Research, Shanghai Institute of Materia Medica, Chinese Academy of Sciences, Shanghai, China

**Keywords:** cynomolgus monkeys, immunoinformatics, MPT64 protein, Mycobacterium tuberculosis, peptide-based indirect ELISA

## Abstract

Tuberculosis (TB) is a severe zoonotic disease that represents a considerable threat to the health of captive non-human primates (NHPs). Conventional serological diagnostic methods often suffer from insufficient sensitivity and specificity, making them difficult to meet the requirements of large-scale screening in monkey colonies. In this study, we predicted and screened Mycobacterium tuberculosis antigenic epitopes using immunoinformatic approaches, and identified a novel antigenic peptide MP71 from the MPT64 protein. Based on the MP71-KLH conjugate, we further developed an indirect ELISA assay. In both the learning cohort (*n* = 19) and independent validation cohort (*n* = 21), the assay exhibited favorable and stable diagnostic performance: in the validation cohort, the AUC was >0.94 (95% CI: 0.8471 to 1.000), with a sensitivity of >92% and a specificity of 100%, together with favorable repeatability. The established ELISA represents a rapid, simple, and reliable serological tool that can be used for routine surveillance and large-scale preliminary screening of tuberculosis in monkeys.

## Graphical Abstract

**Figure:**
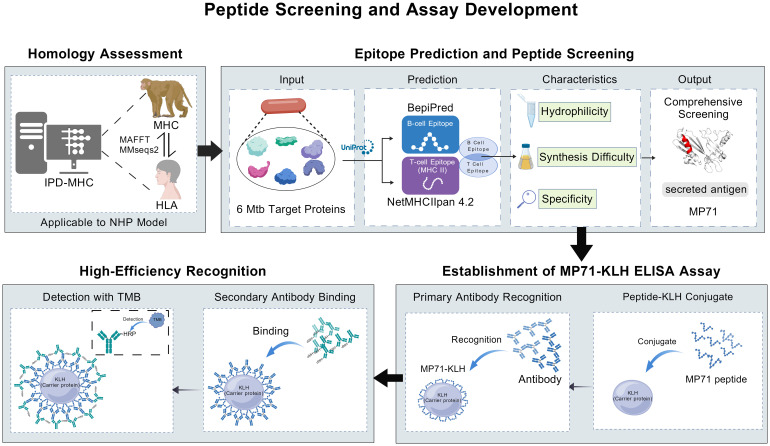
Created with BioGDP.com.

## 1 Introduction

Tuberculosis (TB), a chronic zoonotic disease primarily caused by the *Mycobacterium tuberculosis* complex (MTBC), has a broad host range. Beyond humans, MTBC pathogens can infect cattle, non-human primates (e.g., monkeys), and various wildlife species. TB remains a major global health challenge (Acharya et al., 2020). In 2023, the WHO reported an estimated 10.8 million incident cases worldwide, with approximately 1.2 million fatalities (WHO, 2024). Moreover, in high-burden countries, human TB is spilling over into wildlife populations (Michel et al., 2013). Due to its highly contagious nature in closed settings like captive monkey colonies, early diagnosis of TB is therefore a key intervention for prevention and control (Dara et al., 2016). Conventional TB diagnostic techniques, such as sputum smear, tuberculin skin test, and bacterial culture, are often limited by issues of specificity, speed, and rigorous experimental environment (Ren et al., 2018; Wang et al., 2018). In contrast, serological antibody-based assays offer distinct advantages, such as rapidity, operational simplicity, and not required sophisticated instrumentation (Rasolofo and Chanteau, 1999; Silva et al., 2003). The antigens currently employed in such assays are primarily recombinant proteins (e.g., ESAT-6, CFP-10) (Ma et al., 2020; Rodríguez-Hernández et al., 2020) or specific cell wall molecules (e.g., certain glycolipids and polyketides) (Rahlwes et al., 2023). However, conventional serological assays often suffer from limited specificity (Khalid et al., 2022). For instance, a study from Souza et al. (2019), an ELISA was developed using a recombinant ESAT-6/MPB70/MPB83 chimeric protein as the coating antigen. Against the gold standard of bacterial culture, this assay demonstrated sensitivity of 79.5% and specificity of 75.5%. Additionally, the production of recombinant protein antigens remains costly and time-consuming. To overcome these challenges, it is imperative to selecting novel diagnostic antigens with superior specificity and sensitivity.

However, the discovery of diagnostic antigens often entails inefficient and costly experimental screening of vast candidate libraries. Immunoinformatics addresses this challenge by merging computational predictions with immunological principles, enabling the rapid and focused identification of candidates (Bulla et al., 2025). With the advent of immunoinformatics, screening strategies for novel diagnostic antigens based on computational predictions have been increasingly adopted. Examples include the development of sensitive and specific peptide-based ELISA for *canine visceral leishmaniasis* (Moreira et al., 2024), performance-enhanced assays for parvovirus (Kaur et al., 2024), and early-detection tests for *Anisakis pegreffii* via immunoproteomics integration (Wang et al., 2024). Collectively, these studies demonstrate that immunoinformatics can robustly guide the discovery and development of serodiagnostic antigens with high sensitivity and specificity.

In immunological research, major histocompatibility complex (MHC) molecules play a crucial role in antigen presentation and immune responses. MHC molecules present antigen peptides by interacting with T cell receptors (TCRs), thereby activating T cell responses and initiating the immune response (Lam et al., 2024). Major histocompatibility complex class II (MHC II) molecules, known as HLA-II molecules in humans, are primarily expressed by professional antigen-presenting cells such as dendritic cells, macrophages, and B cells. They are responsible for presenting processed exogenous antigens to CD4^+^ T helper cells (Rocha and Neefjes, 2008). Activated T cells further promote B cell differentiation and antibody production, thereby playing a central role in humoral immunity (Teillaud et al., 2024). The organization of the MHC II region in humans and NHPs is highly similar, providing a crucial foundation for cross-species epitope prediction (Heijmans et al., 2020).

Therefore, building upon existing research confirming structural similarities between human and non-human primate MHC II regions, this study further analyzed amino acid sequence conservation within the core binding region (β1 domain) of the most common allele, DRB. Based on this analysis, a systematic and efficient immunoinformatics strategy was employed to evaluate B-cell and MHC II epitopes within key Mtb targets. To ensure diagnostic specificity, we conducted cross-reactivity risk analysis of candidate peptides against common pathogens in experimental monkeys, significantly reducing the possibility of misdiagnosis. Finally, informed by literature, we overcame the inherent poor adsorption of short peptides and enhanced assay specificity by conjugating them to a carrier protein (Hnasko, 2015; Duan and Zhang, 2017; Milchram et al., 2022; Qiao et al., 2023). Subsequent experimental validation confirmed the favorable diagnostic efficacy of the established assay. Herein, we developed a complete immunoinformatics-driven workflow for screening and validating diagnostic antigenic peptides, culminating in the establishment of a corresponding detection assay.

## 2 Materials and methods

### Peptide and serum samples

Peptide Synthesis: All peptides and peptide-KLH conjugates were synthesized by GenScript Biotech Corporation (Nanjing, China). The conjugation of peptides to KLH was performed using the MBS (m-Maleimidobenzoyl-N-hydroxysuccinimide ester) method, wherein the NHS ester group of MBS reacts with the amino groups of KLH, while the maleimide group reacts with the thiol group of the peptide, thereby achieving directional covalent linkage. Since the peptide sequences used in this study did not contain cysteine residues, an additional Cys residue was introduced at the N−terminus during peptide synthesis. The carrier protein, keyhole limpet hemocyanin (KLH), was sourced from Millipore Sigma.

Sample collection: A panel of 27 serum samples from tuberculosis-confirmed monkeys (diagnosed by tuberculin skin test, TST) was provided by the Guangxi Zhuang Autonomous Region Terrestrial Wildlife Medical-aid and Monitoring Epidemic Diseases Research Center. Negative Serum: 13 negative control serum samples were sourced from the Drug Safety Evaluation Centre of the Zhongshan Institute for Drug Discovery.

Serum Separation: all manipulations were carried out within biological safety cabinet in a designated BSL-2 laboratory. Additionally, all serum samples were inactivated by heat treatment at 56°C for 30 minutes.

### DRB sequence similarity assessment

Full-length human HLA-DRB protein sequences were obtained from the IPD–MHC database (Maccari et al., 2025), together with the corresponding DRB sequences from Macaca fascicularis (MAFA) and Macaca mulatta (MAMU). All sequences were aligned using MAFFT in auto mode (Katoh et al., 2002), with the human DRB1 sequence serving as the reference anchor. Domain annotations from UniProt were used to delineate the β1 domain (amino acids 30–124), which encompasses the principal antigen-binding region of MHC II molecules (Ahmad et al., 2025). The β1 segments were extracted from the alignment to generate a dedicated β1 domain dataset. The β1 domain sequences of the two NHPs were then used as the query set, and the human HLA-DRB β1 domain sequence as the target. Sensitive similarity searches were performed using MMseqs2 to assess interspecies sequence similarity between non-human primate DRB and human HLA-DRB (Kallenborn et al., 2025).

### Epitope prediction and peptide screening

We selected 6 target proteins associated with Mtb infection, including MPT64 (P9WIN9), MMPL3 (P9WJV5), ESAT6 (P9WNK7), PSTS1 (P9WGU1), FTSK (P9WNA3), and RV2209 (P9WLI3). The complete amino acid sequences of these proteins were retrieved from the UniProt database. B-cell epitopes were predicted using BepiPred-2.0 (Jespersen et al., 2017), and MHC II epitopes were predicted using NetMHCIIpan 4.2 (Nilsson et al., 2023), with 15 high-frequency HLA-DRB1 alleles specified as the restriction elements. Both tools were accessed via the DTU Health Tech online platform. Following initial filtering, we derived the intersection between the B-cell epitopes and the HLA-DRB1–restricted MHC II epitopes to identify peptide candidates with both B-cell antigenicity and helper T-cell recognition potential. The hydrophilicity and synthetic feasibility of the intersecting peptides were subsequently evaluated using GenScript’s peptide analysis tool.

### Analysis of peptide specificity and structure

BLAST alignment was performed for the candidate peptides against the UniProt database using a default threshold (E-value < 10) to identify any identical sequences present in organisms outside the MTBC. To assess potential cross-reactivity, we specifically examined whether these peptides were present in pathogens commonly found in laboratory cynomolgus monkeys, based on the pathogen list provided by Suzhou Xishan Biotechnology Co., Ltd.

For structural assessment, experimentally resolved three-dimensional structures of the target proteins were retrieved from the RCSB PDB database. For proteins without available structures, models were generated using AlphaFold3 with default settings (Abramson et al., 2024). The candidate peptides were then mapped onto these structures and visualized using PyMol(version 3.0) to examine their spatial localization.

### Establishment and evaluation of the MP71-KLH detection method

To establish optimal assay conditions, we screened a range of parameters. The MP71-KLH peptide was coated at concentrations of 0.625 to10 μg/mL overnight at 4°C. Simultaneously, secondary antibodies—horseradish peroxidase (HRP)-labeled goat anti-human IgM and HRP-labeled rabbit anti-monkey IgG—were tested at dilutions of 1:2500 to1:20,000. Serum samples were serially diluted at 1:100 to 1:2,700. After washing, color was developed with TMB substrate for 15–20 minutes at 37°C in the dark. The reaction was stopped with 2 M H_2_SO_4_, and the absorbance at 450 nm was measured.

Experimental conditions were optimized via a chessboard titration method. Key parameters screened included: b blocking buffer: 3% BSA-PBST, 5% BSA-PBST, 3% skimmed milk, or 5% skimmed milk.

Using the conditions established through screening, a preliminary validation was performed on a learning cohort of 19 monkey serum samples. Both HRP-conjugated IgG and IgM antibodies were analyzed, followed by the determination of a cut-off value. ROC analysis was conducted for the candidate peptide (MP71-KLH) based on the serum assay results, using GraphPad Prism 9 software.

### Validation of the detection method

To evaluate the sensitivity, we compared the performance of the MP71-KLH peptide (10aa-KLH) and the full-length MPT64 protein (205aa), a pooled serum sample from 6 TB -positive monkeys was prepared. This pooled sample was subjected to serial dilution at ratios of 1:100, 1:300, 1:900, 1:2,700, and 1:8,100. The optical density at 450 nm (OD_450_) for each dilution was then measured under the established assay conditions.

To validate the assay’s specificity, serum samples were obtained from the following cohorts: 6 healthy monkeys, 6 Mtb-infected monkeys, and 6 B virus (BV)-infected monkeys. Furthermore, serum from an influenza-infected mouse was incorporated to assess potential cross-reactivity with antibodies against an unrelated pathogen.

To assess repeatability, 10 selected serum samples (5 TB positive, 5 TB negative) were analyzed in 6 technical replicates under the established conditions. The coefficient of variation (CV%) was calculated for each sample as (standard deviation/mean) × 100%.

## 3 Result

### Homology assessment reveals predominant similarity of primate DRB alleles to human HLA-DRB1

**Figure 1:**
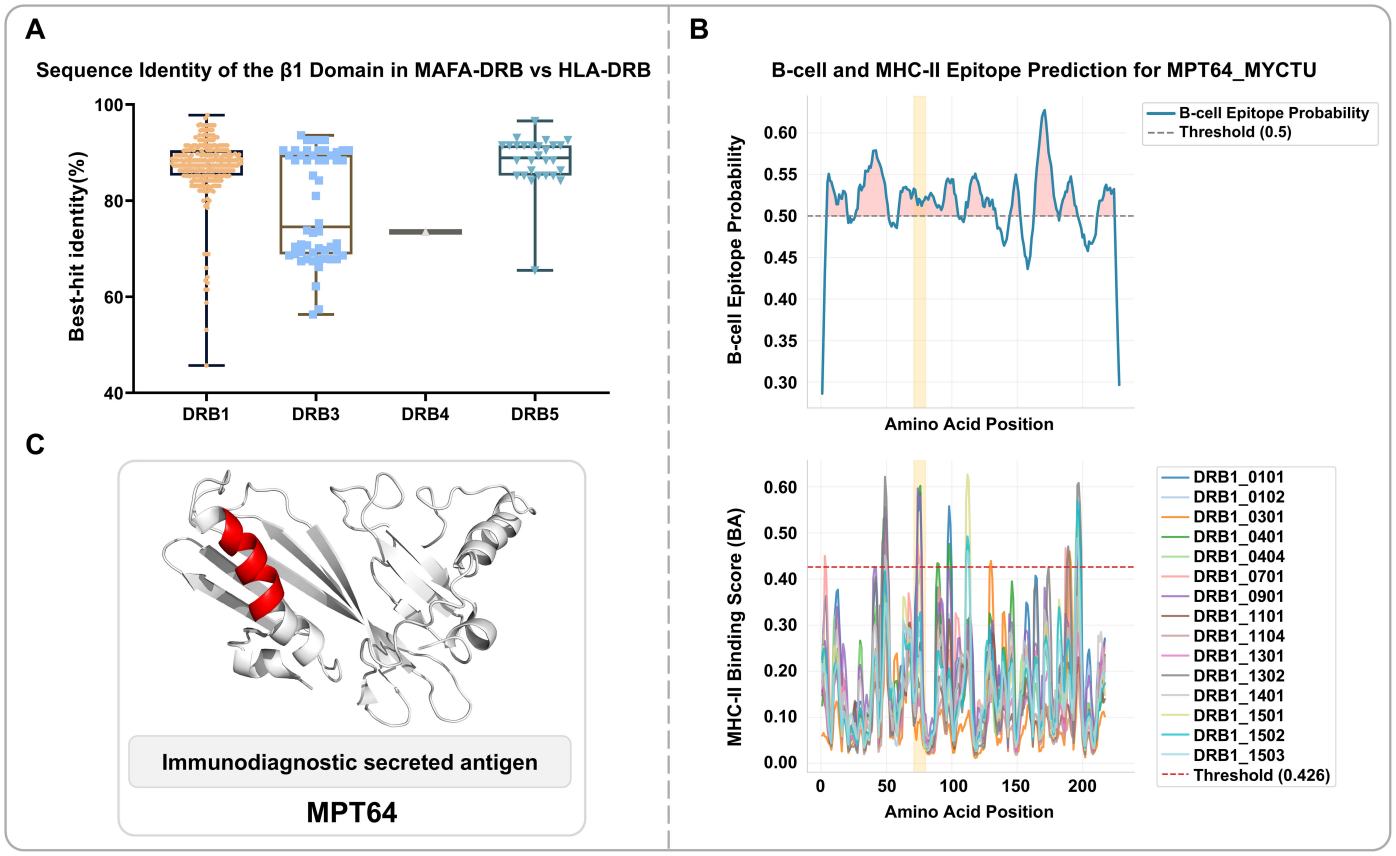
Comprehensive analysis of the candidate epitope peptide MP71 derived from MPT64. **(A)** Sequence homology comparison of the β1 domain between MAFA-DRB alleles and human HLA-DRB. **(B)** Comprehensive epitope prediction profiles of protein MPT64. The upper panel depicts B-cell epitope prediction results, whereas the lower panel depicts MHC-II epitope prediction results. The curve plots the relationship between binding prediction score and amino acid position, with the dashed horizontal line indicating the positive threshold. The candidate peptide region is highlighted in yellow. **(C)** Structural localization of MP71 (highlighted in red) on the MPT64 protein surface.

In developing ELISA assays for detecting TB -specific antibodies in NHPs, attention is typically placed on selecting B-cell epitopes that can be recognized by antibodies (Cyster and Wilson, 2024). However, the generation of robust antigen-specific antibody responses also depends on adequate T-cell help, which in turn requires that antigenic peptides be effectively presented by MHC II molecules (Heyman, 2024). The β1 domain constitutes the core of the peptide-binding cleft of MHC II molecules, where polymorphic residues determine peptide-binding specificity (Stern et al., 1994; Dessen et al., 1997; Smith et al., 1998; Ooi et al., 2017). We focused on DRB genes because among MHC II loci, DRB exhibits the highest polymorphism (Robinson et al., 2003). However, existing prediction software provides comprehensive support for human HLA alleles, while coverage for NHPs remains extremely limited. Thus, we analyzed β1-domain amino acid homology between humans and two non-human primate species. Among 363 MAFA DRB alleles, 280 (77.1%) showed highest similarity to human HLA-DRB1, with an average identity of 87.3%. Fewer alleles matched HLA-DRB3 (*n* = 54, mean identity 81.4%), HLA-DRB5 (*n* = 28, mean identity 88.3%), or HLA-DRB4 (*n* = 1, identity 73.5%). Although DRB5 matches showed slightly higher identity than DRB1, the number of alleles corresponding to DRB5 is significantly lower than that of DRB1 (**Figure 1A**). A similar distribution was observed in MAMU. Among the 280 MAMU DRB alleles analyzed, 213 (76.1%) exhibited the highest sequence similarity to human HLA-DRB1, with an average sequence identity of 87.7%. The number of alleles matching other human DRB genes (DRB3, DRB5, DRB4) was 48, 17, and 2, respectively, with average sequence identity of 80.6%, 87.3%, and 85.9% (**Supplementary Figure 1**). Overall, HLA-DRB1 demonstrated the most consistent and extensive homology across the two primate species, supporting its use as the reference molecule for subsequent MHC II epitope prediction.

### Prediction and selection of immunogenic peptides from key M. tuberculosis proteins

Our targets included secretory proteins (ESAT-6, MPT64), proteins involved in cell division (FtsK) and wall synthesis (Psts1), host interaction factors (MMPL3), and a probable conserved integral membrane protein (Rv2209) (Ernst et al., 2019; Watson et al., 2021; Williams and Abramovitch, 2023; Zhu et al., 2023; Lu et al., 2025). We focused on these key Mtb target proteins and systematically predicted their B-cell epitopes and peptide–MHC II binding affinity. All predictions were performed using BepiPred-2.0 and NetMHCIIpan-4.2. Based on the results of B-cell linear epitopes and peptide–MHC II binding affinity, all proteins were able to screen out peptides that met the criteria (**Supplementary Figure 2-6**). By applying a comprehensive threshold-based screening strategy, we identified 6 candidate peptides that simultaneously exhibit both B-cell and MHC II T-cell epitope features, along with good hydrophilicity and a “normal” synthesis difficulty rating. Among these, peptide MP71 demonstrated potential in subsequent experimental validation (**Figure 1B**). The functional annotations of their source proteins and detailed peptide information are presented in **Supplementary Table 1**. The molecular weights of the selected peptides ranged from 1028.15 to 1707.04 Da.

### Sequence specificity and surface exposure of immunogenic peptide candidates

To evaluate species specificity, a BLAST search against the UniProt database was performed. The results revealed distinct specificity patterns among the peptides. All reported BLAST hits met the default UniProt E-value threshold (E-value < 10). Given the short length of the peptides, E-values were interpreted with caution and were used primarily as an initial filtering criterion rather than a direct measure of specificity. Accordingly, peptide specificity was evaluated mainly based on the number of BLAST hits and their taxonomic distribution. MP71 matched only 6 sequences across all species, all of which originated from the MTBC, indicating its particularly high potential for specific pathogen recognition (**Table 1**). Although peptides such as MM181 and FT238 produced a greater number of BLAST hits and exhibited broader distributions within the Mycobacterium genus, none of the 6 candidate peptides showed detectable homology to proteins from common pathogenic species infecting monkeys, as determined by BLAST analysis (**Supplementary Figure 7**). Importantly, the majority of the homologous sequences identified by BLAST were derived from the Mycobacterium genus. These findings underscore the close association of the candidate peptides with the target pathogen and support their potential as specific serological targets for TB diagnosis.

**Table 1:** Sequence alignment of peptide MP71 across different species via BLAST analysis.

No.	Accession^a^	Protein name	Organism	Identity	E-value^b^
1	P9WIN9	Immunogenic protein MPT64	Mycobacterium tuberculosis (H37Rv)	100%	0.95
2	P9WIN8	Immunogenic protein MPT64	Mycobacterium tuberculosis (CDC1551)		
3	P0A5Q5	Immunogenic protein MPB64	Mycobacterium bovis (AF2122/97)		
4	A0ABV1ME25	Immunoprotective protein MPT64	Mycobacterium canetti		
5	A5U400	Immunogenic protein MPT64	Mycobacterium tuberculosis (H37Ra)		
6	A0ABX2VLF8	Immunogenic protein MPB64	Mycobacterium mungi		

^a^ Accession numbers refer to UniProt protein identifiers.

^b^ E-values reflect the probability of random BLAST matches. Relatively large E-values are expected for short peptide sequences; therefore, peptide specificity was evaluated primarily based on the number and taxonomic distribution of BLAST hits.

Structural analysis confirmed that peptide MP71 is located on the protein surface, indicating high structural accessibility for immune recognition (**Figure 1C**). The remaining peptides also exhibit the same structural characteristics (**Supplementary Figure 8**).

### Establishment and evaluation of the MP71-KLH detection method

We utilized 40 serum samples, allocating 19 to a learning cohort for method development and reserving 21 as an independent validation cohort for final confirmation. In the initial phase, IgM was selected as the primary detection target because its superior performance over IgG. To enhance the assay, we conjugated the antigen to KLH. This strategic modification led to a substantial improvement in the detection performance of IgG when it was re-assessed under the optimized protocol.

Initial screening of 6 candidate peptides (MP71, ES31, Ps111, Ft238, Rv351, Mm181) identified MP71 as the top performer based on its highest P/N ratio, leading to its selection for subsequent assay optimization (**Supplementary Figure 9**). Followed by this, the diagnostic performance of the anti-MP71 IgM assay was evaluated via ROC curve analysis on a set of 13 positive and 6 negative samples. The Youden index was used to calculate an optimal cut-off of 0.6375, at which the assay yielded an AUC of 0.8590, with a sensitivity of 69.23% and a specificity of 100% (**Supplementary Figure 10**).

Although the initial ELISA under these conditions yielded an AUC > 0.8, we sought further optimization. Consistent with literature recommendations for small-molecule peptides (Hnasko, 2015), we conjugated MP71 to the carrier protein Keyhole limpet hemocyanin (KLH). Following conjugation, the resulting MP71-KLH conjugate enabled effective detection with both IgG-HRP and IgM-HRP and was subsequently used as the coating antigen in all following experiments.

**Figure 2:**
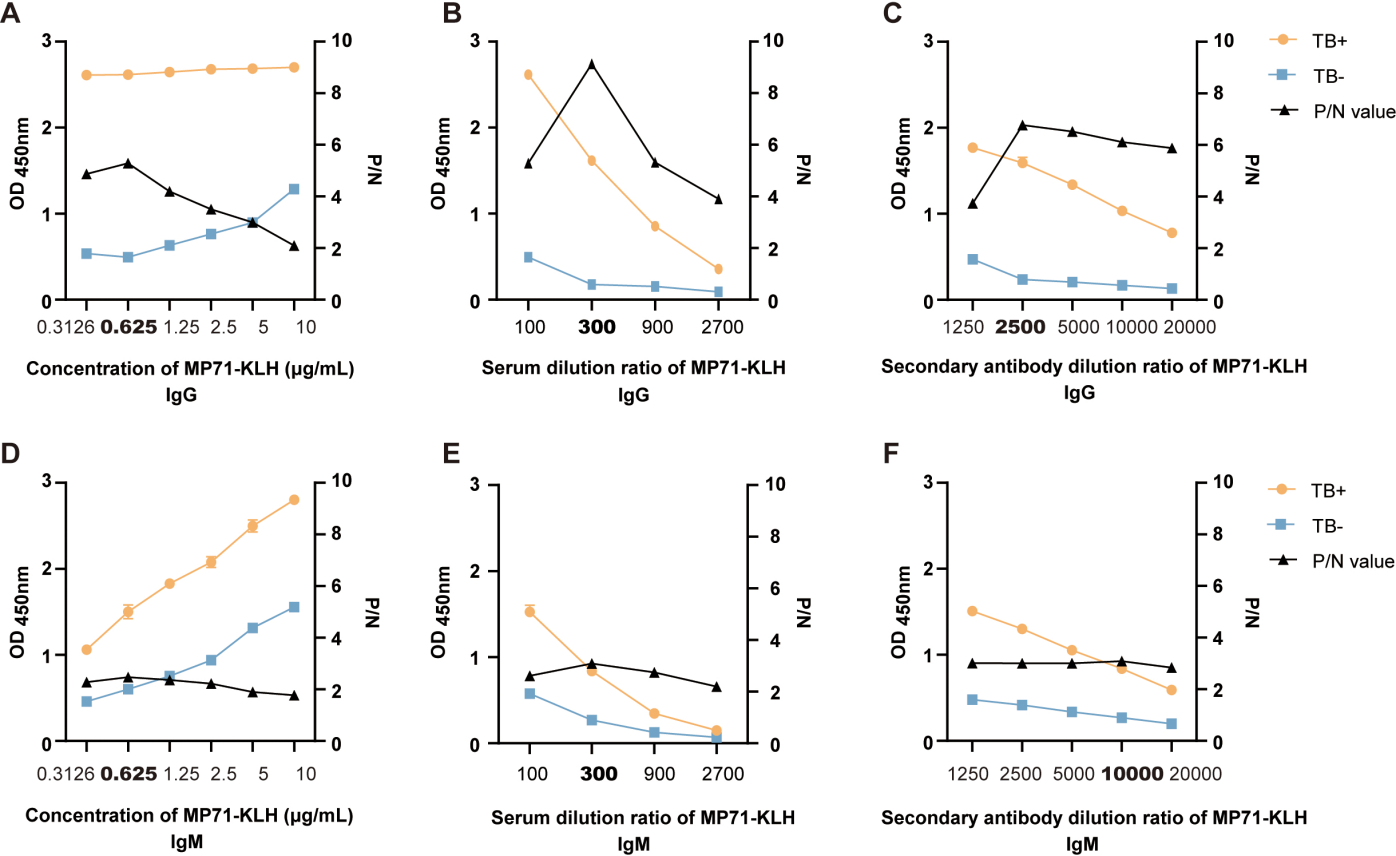
Optimization of ELISA experimental conditions using MP71-KLH conjugates as coating antigens. Two detection systems were utilized: IgG-HRP detection **(A–C)** and IgM-HRP detection **(D–F)**. The checkerboard titration analyses were performed on coating antigen concentration **(A, D)**, serum dilution **(B, E)** and secondary antibody dilution **(C, F)**. Bold numbers indicate the selected optimal conditions.

Optimal conditions were determined by re-screening coating concentration, serum dilution, and secondary antibody dilution. The final parameters were a coating concentration of 0.625μg/mL for MP71-KLH (**Figures 2A, D**) and serum dilutions of 1:300 for detection with IgG-HRP and IgM-HRP, respectively (**Figures 2B, E**). The optimal dilutions for the HRP-conjugated secondary antibodies were determined to be 1:2500 for rabbit anti-monkey IgG and 1:10,000 for goat anti-Human IgM (**Figures 2C, F**), based on the maximal P/N ratio (P/N = [OD_450_ positive]/[OD_450_ negative]).

Subsequently, we screened blocking solutions and identified a buffer containing 5% (w/v) bovine serum albumin (BSA) in PBST as the optimal blocking buffer (**Supplementary Figure 11**).

### Determination the cut-off value

To compare their diagnostic performance, we assessed both IgG and IgM levels in the cohort of 19 monkeys, and then establishment of the cut-off value.

**Figure 3:**
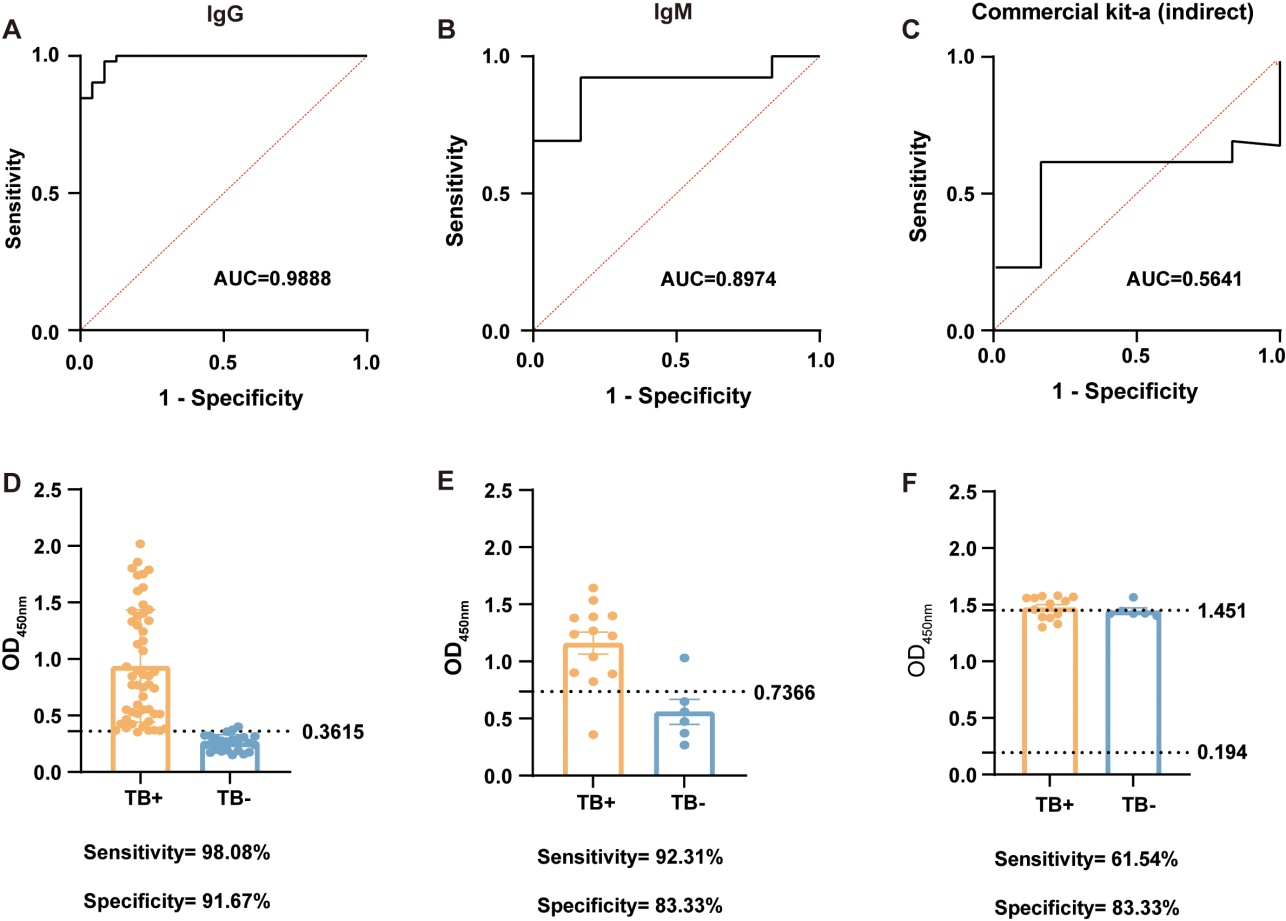
Cut-off determination and diagnostic performance in the learning cohort (*n* = 19). **(A, D)** The present IgG ELISA (4 technical replicates; cut-off = 0.3615). **(B, E)** The present IgM ELISA (cut-off = 0.7365). **(C, F)** Commercial IgG ELISA (kit A). For kit A, diagnostic performance was shown at both the manufacturer-recommended cut-off (0.194) and the Youden-index derived cut-off (1.451); The sensitivity and specificity of the Youden index method were 61.54% and 83.33%, respectively.

ROC analysis revealed that the IgG-HRP conjugate (cut-off: 0.3615) demonstrated superior diagnostic accuracy, We employed a conservative analytical approach to account for technical variability by performing the ROC analysis on all individual data points from the 4 replicate wells (using averaged values of individual biological samples for ROC analysis yielded an AUC of 1.0, owing to the small learning cohort, which would cause over-optimistic estimation), without averaging (AUC = 0.9888, 95% CI: 0.9718 to 1.000, sensitivity: 98.08%, specificity: 91.67%, **Figures 3A, C**) over the IgM-HRP conjugate (AUC = 0.8974, 95% CI: 0.7487 to 1.000, cut-off: 0.7366, sensitivity: 92.31%, specificity: 83.33%, **Figures 3B, E**). Therefore, the cut-off value for seropositivity was set at an OD of 0.3615 (OD ≥ 0.3615 = positive; OD < 0.3615 = negative). The commercial kit (indirect ELISA) performance was evaluated under two cut-off criteria. Using the manufacturer’s definition (negative control mean OD + 0.15), the cut-off was 0.194 (**Figures 3C, F**). In contrast, when we determined the optimal cut-off using Youden index, the value was established at 1.451, at this Youden-derived threshold, the assay yielded five false-positive and one false-negative result, corresponding to a sensitivity of 61.54% and a specificity of 83.33%.

### Diagnostic performance in the validation cohort

We further validated the assay in an independent cohort of 21 cynomolgus monkey serum samples and compared its performance with commercial ELISA. Kit a (indirect ELISA) was used in the learning cohort, as it shares the same detection principle as our established assay for internal comparison and cost-effective exploratory analysis, and Kit b (double-antigen sandwich ELISA) was selected for the validation cohort for high-specificity external confirmatory detection.

**Figure 4:**
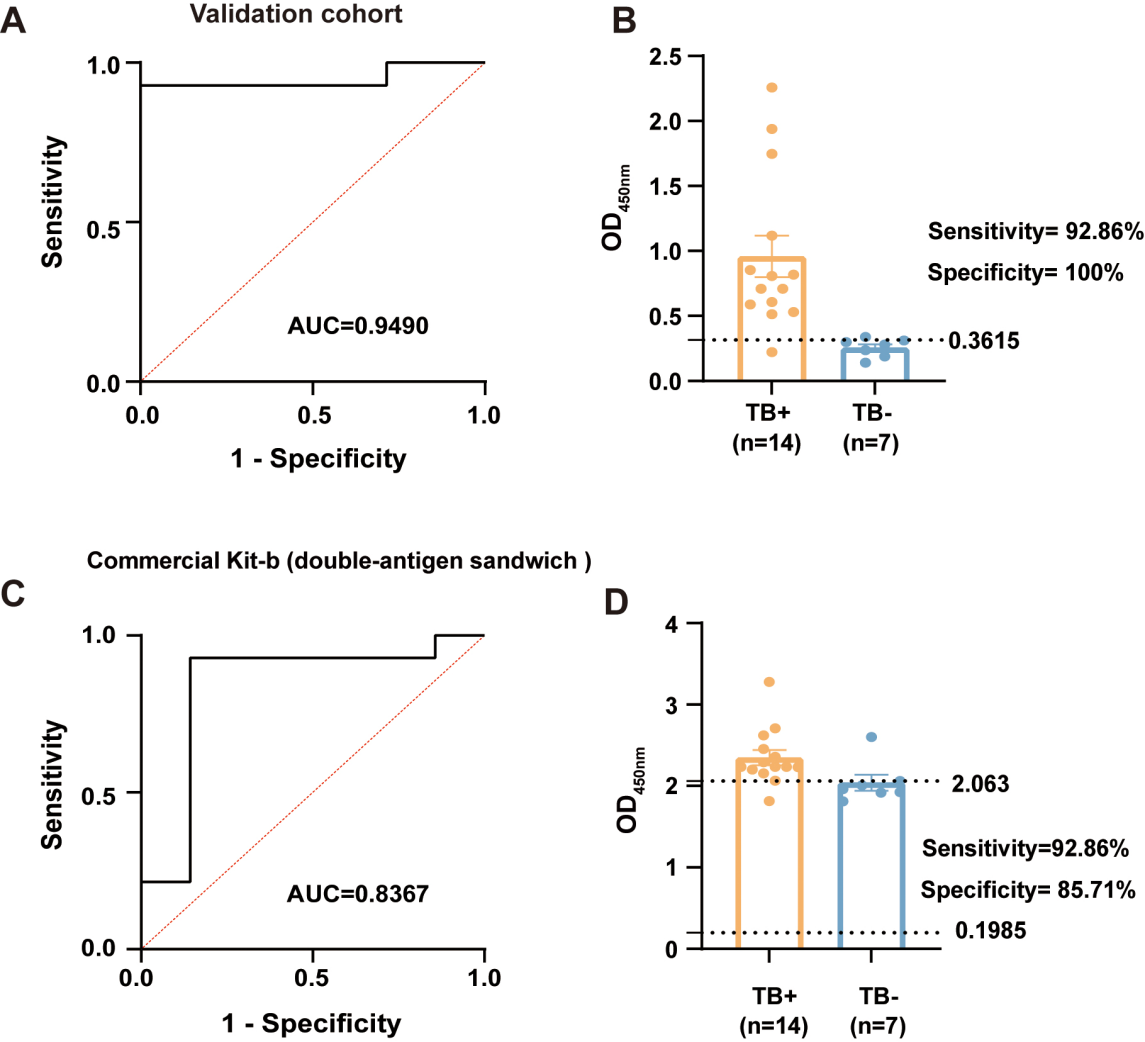
Diagnostic performance in the validation cohort (*n* = 21). **(A, B)** The present IgG ELISA. **(C, D)** Commercial antigen-sandwich IgG ELISA (kit B). For kit B, diagnostic performance was shown at both the manufacturer-recommended cut-off (0.1985) and the Youden-index derived cut-off (2.063). The sensitivity and specificity of the Youden index method were 92.86% and 85.71%, respectively.

This method, which provides a more robust performance estimate, yielded an AUC of 0.9490 (cut-off: 0.3615, 95% CI: 0.8471 to 1.000, sensitivity: 92.81%, specificity: 100%, **Figures 4A, B**).

The commercial kit-B (Antigen Sandwich ELISA) performance was evaluated under two cut-off criteria. Using the manufacturer’s definition (negative control mean OD + 0.15), the cut-off was 0.1985. In contrast, when we determined the optimal cut-off using Youden index, the value was established at 2.063. At this Youden-derived threshold, the assay yielded one false-positive and one false-negative result, corresponding to a sensitivity of 92.86% and a specificity of 85.71%. ROC analysis performed using GraphPad Prism 9 software yielded an area under the curve (AUC) of 0.8367 (**Figures 4C, D**).

### Validation of the detection method

**Figure 5:**
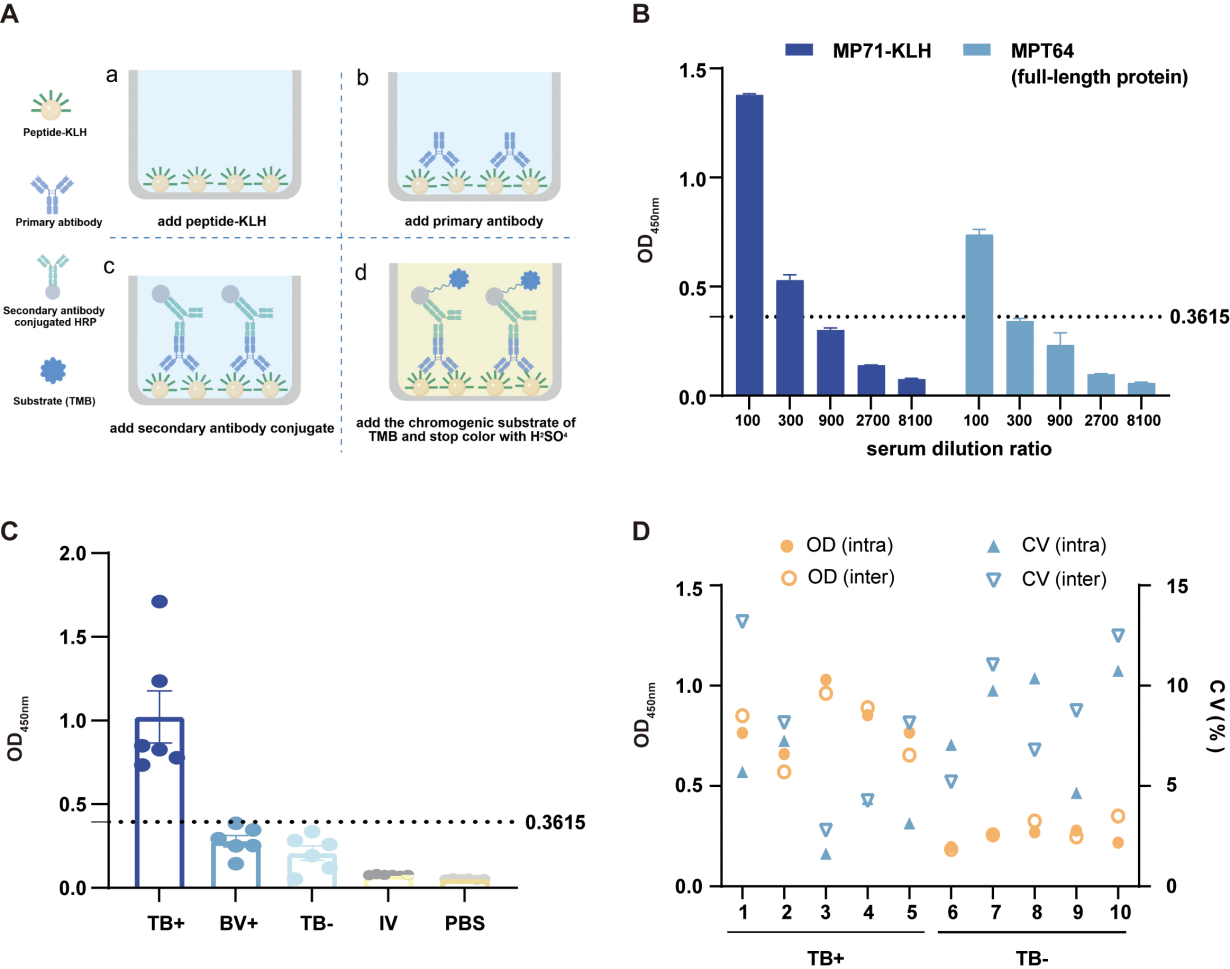
Method validation (Self-developed MP71–KLH conjugate IgG ELISA): specificity, sensitivity and repeatability. **(A)** Schematic diagram of the ELISA workflow. **(B)** Sensitivity check: serum serial dilution. **(C)** Specificity check: TB+ (Tuberculosis-positive monkey), BV+ (Herpes B virus-positive monkey), TB- (Tuberculosis-negative control), IV (influenza mouse), PBS. **(D)** Repeatability: 10 serum samples (5 TB positive, 5 TB negative) tested in duplicate (*n* = 6); intra- and inter-assay CV < 15%.

Sensitivity: the MP71 peptide demonstrated a higher dilution titer than the full-length protein, retaining reactivity above the cut-off (0.3615) at a 1:300 serum dilution compared to 1:100 for the full-length protein (**Figure 5B**).

Specificity: as shown in **Figure 5C**, when samples were classified by the reference standard, all non-tuberculous control sera exhibited OD values below the 0.3615 threshold, with the exception of one sample from a B virus-positive monkey which was slightly elevated. In contrast, all TB -positive monkey serum showed a markedly higher OD value.

Intra-board and inter-board precision verification:we observed that both the intra- and inter-assay CV were below 15% for all 10 serum samples tested (**Figure 5D**, **Table 2**).

**Table 2:** Repeatability test.

Sample	Intra-batch mean ± SD	CV%	Inter-batch mean ± SD	CV%
1	0.7642 ± 0.0437	5.713	0.8495 ± 0.1119	13.17
2	0.6587 ± 0.0478	7.261	0.5713 ± 0.0466	8.152
3	1.0290 ± 0.0168	1.628	0.9622 ± 0.0269	2.798
4	0.8528 ± 0.0372	4.364	0.8907 ± 0.0381	4.273
5	0.7673 ± 0.0241	3.140	0.6543 ± 0.0533	8.140
6	0.2690 ± 0.0099	3.695	0.2573 ± 0.0284	11.04
7	0.2777 ± 0.0288	10.37	0.3595 ± 0.0280	7.790
8	0.2183 ± 0.0102	4.650	0.3252 ± 0.0221	6.786
9	0.2795 ± 0.0300	10.74	0.2470 ± 0.0216	8.755
10	0.3292 ± 0.0118	3.590	0.3503 ± 0.0437	12.48

## 4 Discussion

This study established a systematic antigen screening strategy based on a “computational priority, experimental validation” pipeline. Using tools such as NetMHCIIpan and BepiPred, we screened the Mtb antigen to identify high-potential peptides, thereby reducing the resource intensity and empirical nature of conventional screening. From multiple candidate proteins, peptide MP71 (derived from MPT64) was successfully identified and validated. An indirect ELISA developed with MP71 demonstrated favorable diagnostic performance in an independent validation cohort (*n* = 21), with an area under the curve (AUC) > 0.94, sensitivity of 92.81%, and specificity of 100%. Notably, the assay requires an exceptionally low coating concentration (0.625 μg/mL), conferring a significant cost advantage over traditional protein antigens and commercial kits. This establishes a solid foundation for its application in large-scale primate screening and holds practical significance for disease surveillance in resource-limited settings.

MPT64, from which MP71 is derived, is encoded in the Region of Difference 2 (RD2, gene Rv1980c) of Mtb H37Rv. This locus confers high specificity to the MTBC (Magalhães et al., 2021) and is associated with strong immunogenicity and diagnostic utility (Cao et al., 2021), providing a foundation for its use as a specific diagnostic target. Experimentally, the MP71-KLH conjugate effectively detected specific IgG antibodies in sera from infected monkeys and showed no cross-reactivity with control sera from monkeys infected with B virus or mice infected with influenza virus, indicating good diagnostic specificity. Furthermore, conjugation to KLH notably enhanced the IgG reactivity of the short peptide, effectively overcoming its limitation of poor adsorption in indirect ELISA formats.

The diagnostic efficacy of strategies analogous to ours has been substantiated in multiple infectious disease models (Souza et al., 2019; Moreira et al., 2024; Bulla et al., 2025), underscoring the broader potential of immunoinformatics for pioneering the development of novel ELISA antigens. However, we recognize certain inherent limitations of immunoinformatics in practical applications. First, the accuracy of epitope prediction is constrained by the coverage of the training datasets for the underlying algorithms (Cao et al., 2021), which can lead to both false-positive and false-negative results. In this study, although we identified 6 candidate peptides exhibiting both B-cell and T-cell epitope characteristics through an integrated prediction strategy, only one peptide MP71 ultimately demonstrated superior immunogenicity in experiments and was subsequently utilized for method development. Second, the high diversity of HLA alleles poses a challenge to the universality of predictive results. We have validated the high homology between humans and monkeys through MHC sequence alignment, thereby supporting the validity of predictions under the human HLA model. However, it is important to note that existing models are primarily constructed based on common HLA subtypes found in human populations. Their applicability may be limited in NHPs or other species. This strategy should be used with caution for cross-species predictions, particularly in hosts with significant MHC differences, where it may lead to prediction bias. Furthermore, there is currently a lack of unified standards among different prediction platforms regarding parameter settings, scoring systems, and threshold selection (Watson et al., 2021), which has a certain impact on the comparability and reproducibility of results. Therefore, computational prediction is more suitable as an auxiliary tool for candidate antigen screening and experimental decision-making rather than a substitute for experimental validation.

Despite the favorable preliminary performance of the established ELISA in the present study, this study has several limitations: due to the scarcity of TB positive monkey samples, the diagnostic performance of the established assay requires further validation in larger-scale studies; although specificity was evaluated using sera from TB negative, B virus positive, and influenza virus-infected animals, the cross-reactivity control panel remains limited, and additional validation against non-tuberculous mycobacteria (NTM) and other common non-human primate pathogens will be conducted with more available samples. Additionally, both the learning and validation cohorts were derived from the same population, which may restrict the generalizability of the findings, necessitating future multi-center, geographically diverse, and independently blinded studies to enhance the assay’s robustness and external applicability. The TST remains the most practical method for large-scale screening in captive monkeys, although it is not a gold standard for tuberculosis confirmation, and subsequent validation may incorporate culture, PCR, or interferon-gamma release assays (IGRA) to improve reliability.

Nevertheless, these limitations also highlight important directions for further optimization and validation of the present assay. given the extreme rarity of TB-positive NHPs samples, the independently validated results of this study can be regarded as reliable preliminary evidence. Notably, the diagnostic performance of serological assays is affected by host immune status and infection stage. This phenomenon is especially prominent for IgM antibodies, whose levels fluctuate and gradually decrease during disease progression, whereas IgG antibodies remain relatively stable. Accordingly, future research will further develop and optimize an IgM-specific diagnostic method based on the currently established IgG detection platform. Assay performance will also be improved through optimization of conjugation strategies or the use of longer peptide fragments. Combining the present method with the above-mentioned techniques is expected to yield a more robust and reliable diagnostic strategy.

In conclusion, this study preliminarily developed and validated a novel peptide antigen, MP71-KLH. The candidate antigen shows promising diagnostic performance, relatively low production cost, and favorable specificity, making it a potential alternative tool for TB surveillance in NHPs. In addition, the immune informatics-driven systematic screening strategy established in this work provides a reference for the rational design of diagnostic antigens against other zoonotic and infectious diseases.

## Data Availability

The original contributions presented in the study are included in the article/**Supplementary Material**. Further inquiries can be directed to the corresponding authors.

## References

[B1] Abramson J. Adler J. Dunger J. Evans R. Green T. Pritzel A. . (2024). Accurate structure prediction of biomolecular interactions with AlphaFold 3. Nature 630, 493–500. doi: 10.1038/s41586-024-07487-w 38718835 PMC11168924

[B2] Acharya B. Acharya A. Gautam S. Ghimire S. P. Mishra G. Parajuli N. . (2020). Advances in diagnosis of tuberculosis: an update into molecular diagnosis of Mycobacterium tuberculosis. Mol. Biol. Rep. 47, 4065–4075. doi: 10.1007/s11033-020-05413-7 32248381

[B3] Ahmad S. Jose da Costa Gonzales L. Bowler-Barnett E. H. Rice D. L. Kim M. Wijerathne S. . (2025). The UniProt website API: facilitating programmatic access to protein knowledge. Nucleic Acids Res. 53, W547–W553. doi: 10.1093/nar/gkaf394 40331428 PMC12230682

[B4] Bulla A. C. S. Sbano da Silva A. Prado Sereno B. Dias M. F. R. Leal da Silva M. (2025). Computational methods in immunoinformatics: Epitope discovery and diagnostic applications. ACS Omega 10, 44816–44839. doi: 10.1021/acsomega.5c05538 41078745 PMC12508925

[B5] Cao X. J. Li Y. P. Wang J. Y. Zhou J. Guo X. G. (2021). MPT64 assays for the rapid detection of Mycobacterium tuberculosis. BMC Infect. Dis. 21, 336. doi: 10.1186/s12879-021-06022-w 33838648 PMC8035777

[B6] Cyster J. G. Wilson P. C. (2024). Antibody modulation of B cell responses-incorporating positive and negative feedback. Immunity 57, 1466–1481. doi: 10.1016/j.immuni.2024.06.009 38986442 PMC11257158

[B7] Dara M. Solovic I. Sotgiu G. D'Ambrosio L. Centis R. Goletti D. . (2016). Call for urgent actions to ensure access to early diagnosis and care of tuberculosis among refugees: Statement of the European Respiratory Society and the European Region of the International Union Against Tuberculosis and Lung Disease. Eur. Respir. J. 47, 1345–1347. doi: 10.1183/13993003.00377-2016 27009169

[B8] Dessen A. Lawrence C. M. Cupo S. Zaller D. M. Wiley D. C. (1997). X-ray crystal structure of HLA-DR4 (DRA*0101, DRB1*0401) complexed with a peptide from human collagen II. Immunity 7, 473–481. doi: 10.1016/s1074-7613(00)80369-6 9354468

[B9] Duan Q. Zhang W. (2017). Genetic fusion protein 3×STa-ovalbumin is an effective coating antigen in ELISA to titrate anti-STa antibodies. Microbiol. Immunol. 61, 251–257. doi: 10.1111/1348-0421.12494 28561305

[B10] Ernst J. D. Cornelius A. Bolz M. (2019). Dynamics of Mycobacterium tuberculosis Ag85B revealed by a sensitive enzyme-linked immunosorbent assay. mBio 10, e00611-19. doi: 10.1128/mbio.00611-19 31015327 PMC6479003

[B11] Heijmans C. M. C. de Groot N. G. Bontrop R. E. (2020). Comparative genetics of the major histocompatibility complex in humans and nonhuman primates. Int. J. Immunogenet. 47, 243–260. doi: 10.1111/iji.12490 32358905

[B12] Heyman B. (2024). Antibody feedback regulation. Immunol. Rev. 328, 126–142. doi: 10.1111/imr.13377 39180190 PMC11659925

[B13] Hnasko R. M. (2015). The biochemical properties of antibodies and their fragments. Methods Mol. Biol. (Clifton NJ) 1318, 1–14. doi: 10.1007/978-1-4939-2742-5_1 26160559

[B14] Jespersen M. C. Peters B. Nielsen M. Marcatili P. (2017). BepiPred-2.0: improving sequence-based B-cell epitope prediction using conformational epitopes. Nucleic Acids Res. 45, W24–W29. doi: 10.1093/nar/gkx346 28472356 PMC5570230

[B15] Kallenborn F. Chacon A. Hundt C. Sirelkhatim H. Didi K. Cha S. . (2025). GPU-accelerated homology search with MMseqs2. Nat. Methods 22, 2024–2027. doi: 10.1038/s41592-025-02819-8 40968302 PMC12510879

[B16] Katoh K. Misawa K. Kuma K. Miyata T. (2002). MAFFT: a novel method for rapid multiple sequence alignment based on fast Fourier transform. Nucleic Acids Res. 30, 3059–3066. doi: 10.1093/nar/gkf436 12136088 PMC135756

[B17] Kaur C. Asrith K. P. Ramachandra S. G. Hegde N. R. (2024). Immunoinformatics-guided recombinant polypeptide-based enzyme-linked immunosorbent assay for seromonitoring of laboratory animals for minute virus of mice and Kilham rat virus. PloS One 19, e0298742. doi: 10.1371/journal.pone.0298742 38412152 PMC10898725

[B18] Khalid H. van Hooij A. Connelley T. K. Geluk A. Hope J. C. (2022). Protein levels of pro-inflammatory cytokines and chemokines as biomarkers of Mycobacterium bovis infection and BCG vaccination in cattle. Pathog. (Basel Switzerland) 11, 738. doi: 10.3390/pathogens11070738 35889984 PMC9320177

[B19] Lam N. Lee Y. Farber D. L. (2024). A guide to adaptive immune memory. Nat. Rev. Immunol. 24, 810–829. doi: 10.1038/s41577-024-01040-6 38831162

[B20] Lu Z. Zhang Y. Zhong Y. Qiang L. Ge P. Lei Z. . (2025). A bacterial effector manipulates host lysosomal protease activity-dependent plasticity in cell death modalities to facilitate infection. PNAS 122, e2406715122. doi: 10.1073/pnas.2406715122 39964716 PMC11874418

[B21] Ma Z. Ji X. Yang H. He J. Zhang Q. Wang Y. . (2020). Screening and evaluation of Mycobacterium tuberculosis diagnostic antigens. Eur. J. Clin. Microbiol. Infect. Dis. Off. Publ. Eur. Soc. Clin. Microbiol. 39, 1959–1970. doi: 10.1007/s10096-020-03951-3 32548683

[B22] Maccari G. Robinson J. Barker D. J. Yates A. D. Hammond J. A. Marsh S. G. E. (2025). The 2024 IPD-MHC database update: a comprehensive resource for major histocompatibility complex studies. Nucleic Acids Res. 53, D457–D461. doi: 10.1093/nar/gkae932 39436012 PMC11701557

[B23] Magalhães C. G. Moreira G. Ferreira M. R. A. Santos L. M. D. Finger P. F. Ramos D. F. . (2021). Novel phage display-derived recombinant antibodies recognizing both MPT64 native and mutant (63-bp deletion) are promising tools for tuberculosis diagnosis. Biologicals J. Int. Assoc. Biol. Standardization 72, 54–57. doi: 10.1016/j.biologicals.2021.07.002 34247914

[B24] Michel A. L. Hlokwe T. M. Espie I. W. van Zijll Langhout M. Koeppel K. Lane E. (2013). Mycobacterium tuberculosis at the human/wildlife interface in a high TB burden country. Transboundary Emerging Dis. 60, 46–52. doi: 10.1111/tbed.12099 24171848

[B25] Milchram L. Soldo R. Regele V. Schönthaler S. Degeorgi M. Baumgartner S. . (2022). A novel click chemistry-based peptide ELISA protocol: development and technical evaluation. BioTechniques 72, 134–142. doi: 10.2144/btn-2021-0107 35234537

[B26] Moreira G. Maia R. Soares N. Ostolin T. Coura-Vital W. Aguiar-Soares R. . (2024). Synthetic peptides selected by immunoinformatics as potential tools for the specific diagnosis of canine visceral leishmaniasis. Microorganisms 12, 906. doi: 10.3390/microorganisms12050906 38792746 PMC11123790

[B27] Nilsson J. B. Kaabinejadian S. Yari H. Peters B. Barra C. Gragert L. . (2023). Machine learning reveals limited contribution of trans-only encoded variants to the HLA-DQ immunopeptidome. Commun. Biol. 6, 442. doi: 10.1038/s42003-023-04749-7 37085710 PMC10121683

[B28] Ooi J. D. Petersen J. Tan Y. H. Huynh M. Willett Z. J. Ramarathinam S. H. . (2017). Dominant protection from HLA-linked autoimmunity by antigen-specific regulatory T cells. Nature 545, 243–247. doi: 10.1038/nature22329 28467828 PMC5903850

[B29] Qiao D. Wu L. Gu C. Shao H. Yao Y. Qin A. . (2023). Establishment and application of a VP3 antigenic domain-based peptide ELISA for the detection of antibody against goose plague virus infection. Front. Microbiol. 14, 1309807. doi: 10.3389/fmicb.2023.1309807 38075886 PMC10701384

[B30] Rahlwes K. C. Dias B. R. S. Campos P. C. Alvarez-Arguedas S. Shiloh M. U. (2023). Pathogenicity and virulence of Mycobacterium tuberculosis. Virulence 14, 2150449. doi: 10.1080/21505594.2022.2150449 36419223 PMC9817126

[B31] Rasolofo V. Chanteau S. (1999). Field evaluation of rapid tests for tuberculosis diagnosis. J. Clin. Microbiol. 37, 4201. doi: 10.1128/jcm.37.12.4201-4201.1999 10636726 PMC85929

[B32] Ren N. JinLi J. Chen Y. Zhou X. Wang J. Ge P. . (2018). Identification of new diagnostic biomarkers for Mycobacterium tuberculosis and the potential application in the serodiagnosis of human tuberculosis. Microb. Biotechnol. 11, 893–904. doi: 10.1111/1751-7915.13291 29952084 PMC6116745

[B33] Robinson J. Waller M. J. Parham P. de Groot N. Bontrop R. Kennedy L. J. . (2003). IMGT/HLA and IMGT/MHC: sequence databases for the study of the major histocompatibility complex. Nucleic Acids Res. 31, 311–314. doi: 10.1093/nar/gkg070 12520010 PMC165517

[B34] Rocha N. Neefjes J. (2008). MHC class II molecules on the move for successful antigen presentation. EMBO J. 27, 1–5. doi: 10.1038/sj.emboj.7601945 18046453 PMC2206127

[B35] Rodríguez-Hernández E. Quintas-Granados L. I. Flores-Villalva S. Cantó-Alarcón J. G. Milián-Suazo F. (2020). Application of antigenic biomarkers for Mycobacterium tuberculosis. J. Zhejiang Univ. Sci. B. 21, 856–870. doi: 10.1631/jzus.B2000325 33150770 PMC7670104

[B36] Silva V. M. Kanaujia G. Gennaro M. L. Menzies D. (2003). Factors associated with humoral response to ESAT-6, 38 kDa and 14 kDa in patients with a spectrum of tuberculosis. Int. J. Tuberculosis Lung Dis. Off. J. Int. Union Against Tuberculosis Lung Dis. 7, 478–484. 12757050

[B37] Smith K. J. Pyrdol J. Gauthier L. Wiley D. C. Wucherpfennig K. W. (1998). Crystal structure of HLA-DR2 (DRA*0101, DRB1*1501) complexed with a peptide from human myelin basic protein. J. Exp. Med. 188, 1511–1520. doi: 10.1084/jem.188.8.1511 9782128 PMC2213406

[B38] Souza I. I. F. Rodrigues R. A. Gonçalves Jorge K. S. Silva M. R. Lilenbaum W. Vidal C. E. S. . (2019). ELISA using a recombinant chimera of ESAT-6/MPB70/MPB83 for Mycobacterium bovis diagnosis in naturally infected cattle. J. Veterinary Med. Sci. 81, 9–14. doi: 10.1292/jvms.18-0364 30305467 PMC6361649

[B39] Stern L. J. Brown J. H. Jardetzky T. S. Gorga J. C. Urban R. G. Strominger J. L. . (1994). Crystal structure of the human class II MHC protein HLA-DR1 complexed with an influenza virus peptide. Nature 368, 215–221. doi: 10.2210/pdb1dlh/pdb 8145819

[B40] Teillaud J. L. Houel A. Panouillot M. Riffard C. Dieu-Nosjean M. C. (2024). Tertiary lymphoid structures in anticancer immunity. Nat. Rev. Cancer 24, 629–646. doi: 10.1038/s41568-024-00728-0 39117919

[B41] Wang S. Wu J. Chen J. Gao Y. Zhang S. Zhou Z. . (2018). Evaluation of Mycobacterium tuberculosis-specific antibody responses for the discrimination of active and latent tuberculosis infection. Int. J. Infect. Dis. IJID Off. Publ. Int. Soc. For. Infect. Dis. 70, 1–9. doi: 10.1016/j.ijid.2018.01.007 29410147

[B42] Wang X. Zeng M. Cheng G. (2024). Immunoproteomic and immunoinformatic approaches identify sensitive antigens for diagnosing Anisakis pegreffii infection. ACS Infect. Dis. 10, 4360–4368. doi: 10.1021/acsinfecdis.4c00708 39495078

[B43] Watson A. Li H. Ma B. Weiss R. Bendayan D. Abramovitz L. . (2021). Human antibodies targeting a Mycobacterium transporter protein mediate protection against tuberculosis. Nat. Commun. 12, 602. doi: 10.1038/s41467-021-20930-0 33504803 PMC7840946

[B44] WHO . (2024). Global Tuberculosis Report 2024. Available online at: https://iris.who.int/handle/10665/379339 (Accessed June 2, 2026).

[B45] Williams J. T. Abramovitch R. B. (2023). Molecular mechanisms of MmpL3 function and inhibition. Microbial Drug Resistance (Larchmont NY) 29, 190–212. doi: 10.1089/mdr.2021.0424 36809064 PMC10171966

[B46] Zhu C. Yang T. Yin J. Jiang H. Takiff H. E. Gao Q. . (2023). The global success of Mycobacterium tuberculosis modern Beijing family is driven by a few recently emerged strains. Microbiol. Spectr. 11, e0333922. doi: 10.1128/spectrum.03339-22 37272796 PMC10434187

