# Supplementary material for: From immunoinformatics insights to assay development: establishment of a peptide-based indirect elisa for cynomolgus tuberculosis detection
Source: Front Cell Infect Microbiol. 2026 Jun 19;16:1799877. doi: 10.3389/fcimb.2026.1799877 (PMC13328505; doi:10.3389/fcimb.2026.1799877)
Supplement: Supplementary file 1 [file DataSheet1.pdf]

Supplemental Table 1

Table S1 Protein functions and epitope details

Protein	Function	Peptide Name	Start-end	HLA-restriction	B-Cell epitope	Molecular Weight
MPT64 (Rv1980c)	Secreted antigen	MP71	71-80	DRB1_0101<br>DRB1_0401<br>DRB1_0701<br>DRB1_0901	71-80	1136.27
ESAT-6 (Rv3875)	RD1 early secretory antigen target 6	ES31	31-40	DRB1_0101	31-40	1074.24
Psts1 (Rv0934)	Phosphate transporter	Ps111	111-120	DRB1_0101<br>DRB1_0404<br>DRB1_0901<br>DRB1_1302	111-120	1028.15
FtSK (Rv2748c)	DNA transport protein	Ft238	238-252	DRB1_0101<br>DRB1_0102<br>DRB1_0701<br>DRB1_0901<br>DRB1_1104<br>DRB1_1301<br>DRB1_1501<br>DRB1_1502<br>DRB1_1503	239-245	1707.04
Rv2209	Probable conserved integral membrane protein	R351	351-359	DRB1_0101<br>DRB1_0404	355-359	1076.22
MmpL3 (Rv0206c)	Essential inner membrane lipid (TMM) transporter	Mm181	181-190	DRB1_0101<br>DRB1_0901	181-188	1212.48

Table S1. Protein functions and epitope details. All peptides are hydrophilic and normal synthesis difficulty.

Fig. S1

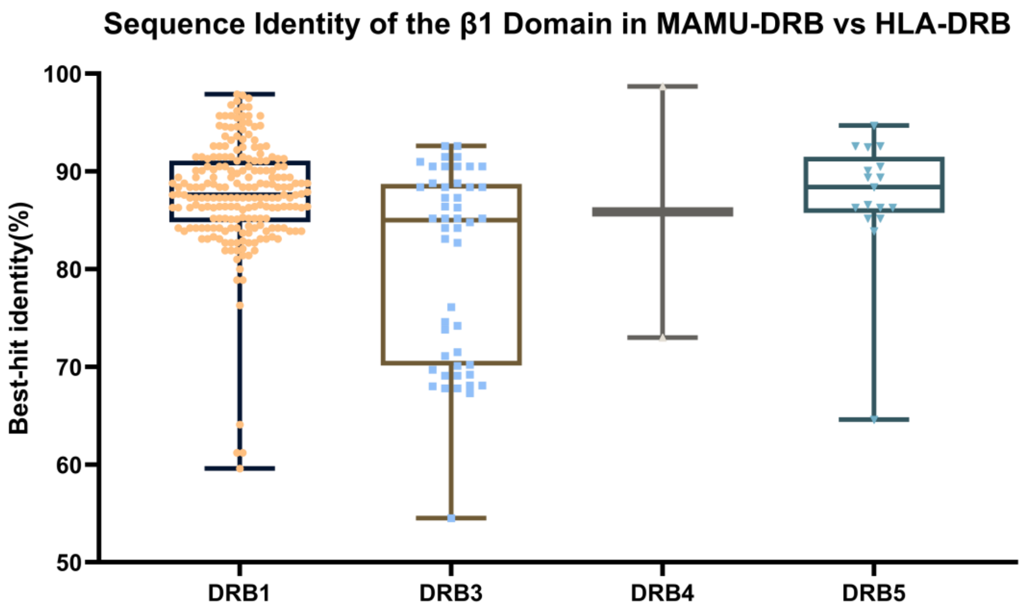

Figure S1. Sequence homology comparison of the β1 domain between MAMU DRB alleles and human HLA-DRB.The box plot illustrates the distribution of percentage amino acid identity for MAMU DRB β1-domain sequences (n=280) to their best-matched human HLA-DRB homolog, grouped by the matched human locus (DRB1, DRB3, DRB4, DRB5).

Fig. S2

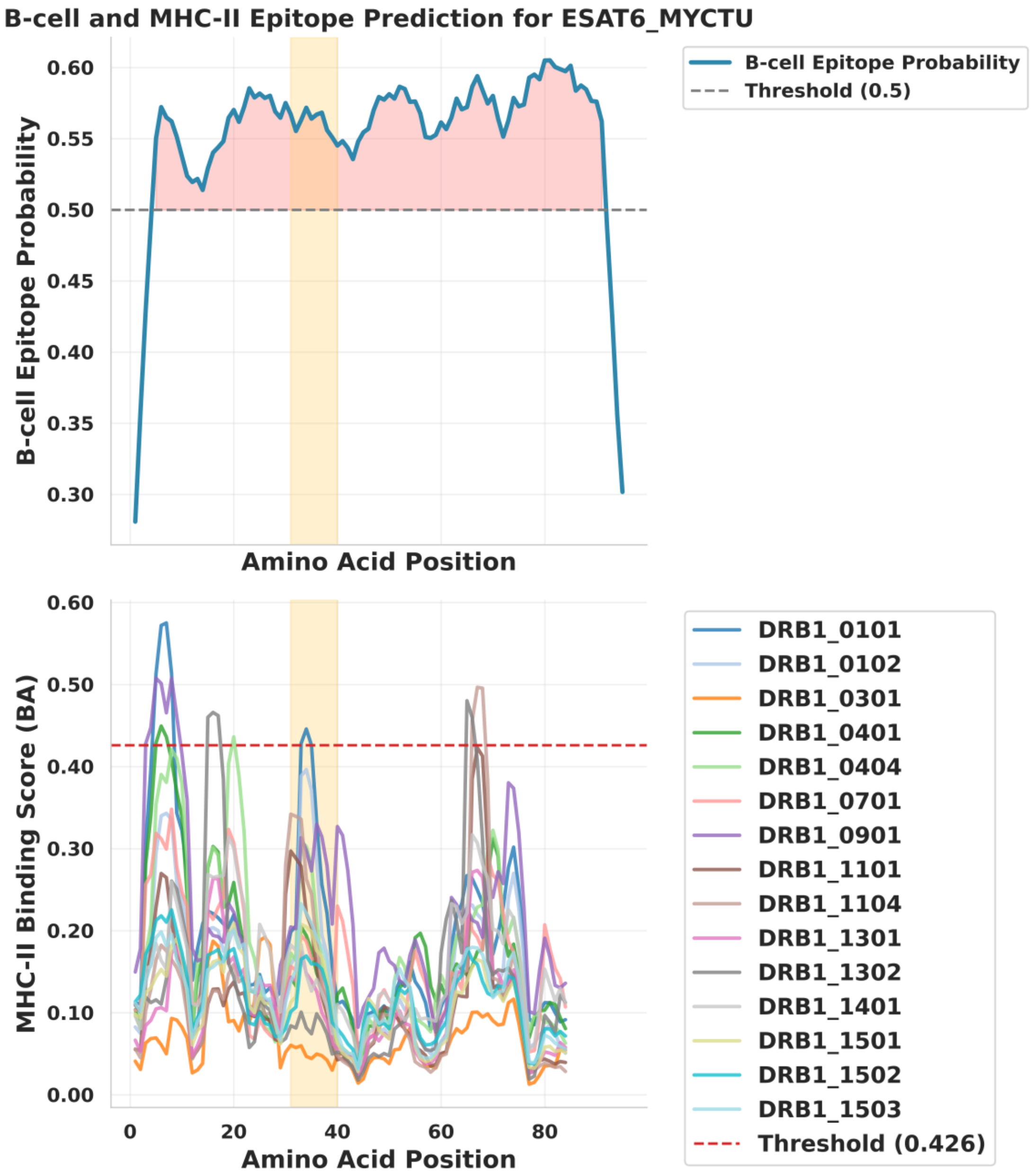

Figure S2. Epitope prediction profile for protein ESAT-6. B-cell (upper) and MHC-II (lower) epitope prediction scores are plotted along the full protein sequence. The dashed line indicates the positive threshold. The candidate peptide region is highlighted in yellow.

Fig. S3

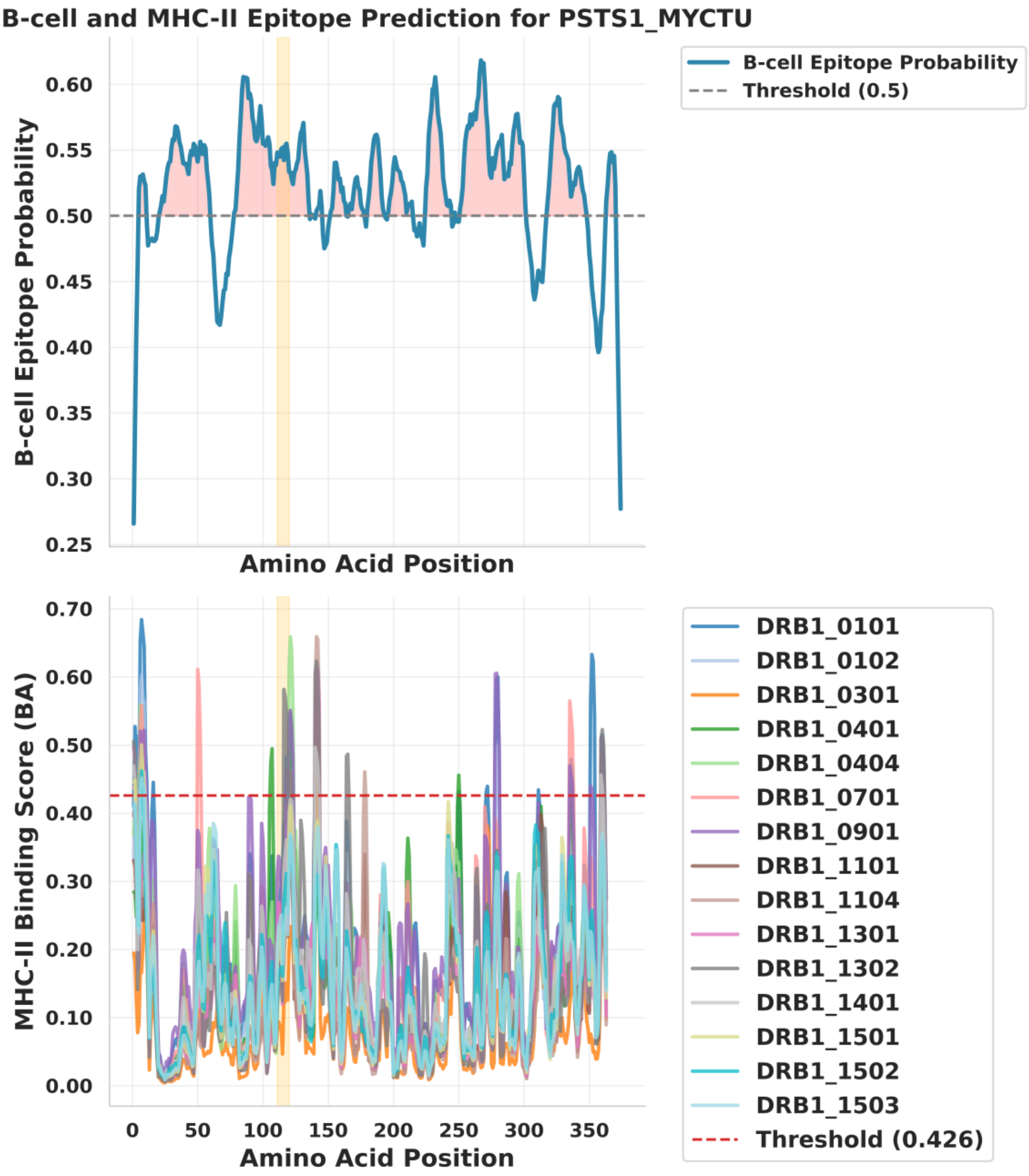

Figure S3. Epitope prediction profile for protein Psts1. B-cell (upper) and MHC-II (lower) epitope prediction scores are plotted along the full protein sequence. The dashed line indicates the positive threshold. The candidate peptide region is highlighted in yellow.

Fig. S4

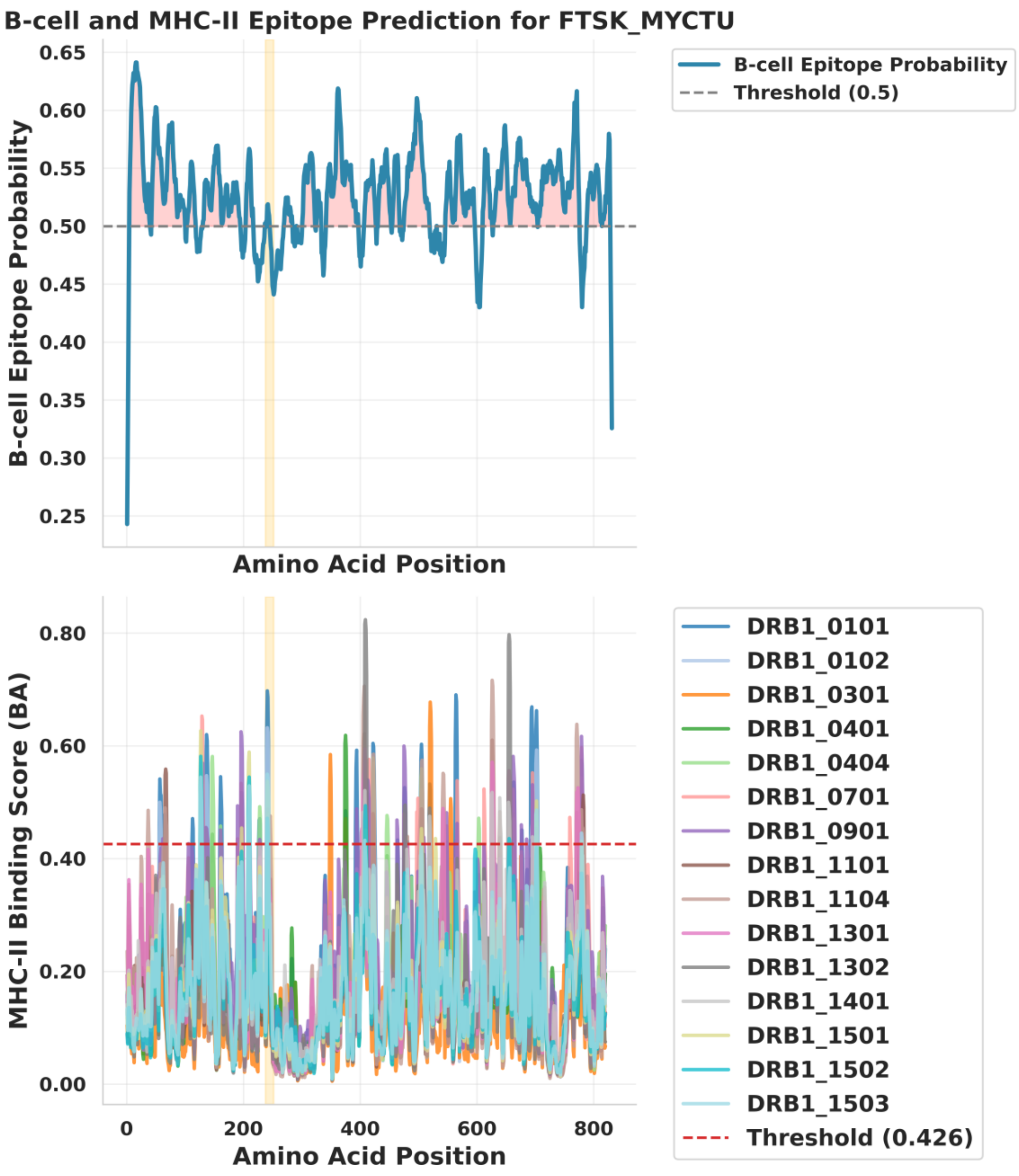

Figure S4. Epitope prediction profile for protein FtSK. B-cell (upper) and MHC-II (lower) epitope prediction scores are plotted along the full protein sequence. The dashed line indicates the positive threshold. The candidate peptide region is highlighted in yellow.

Fig. S5

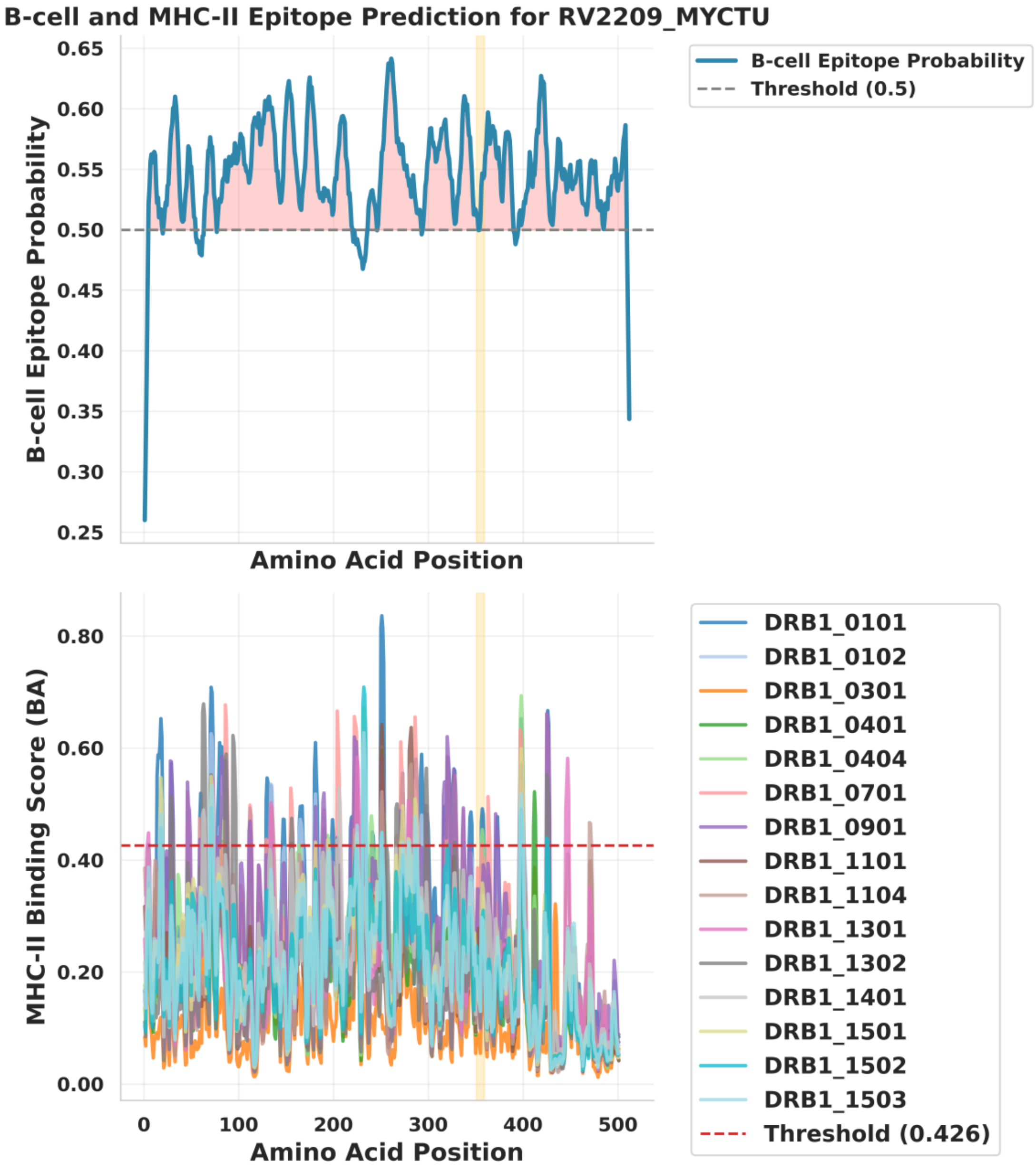

Figure S5. Epitope prediction profile for protein Rv2209. B-cell (upper) and MHC-II (lower) epitope prediction scores are plotted along the full protein sequence. The dashed line indicates the positive threshold. The candidate peptide region is highlighted in yellow.

Fig. S6

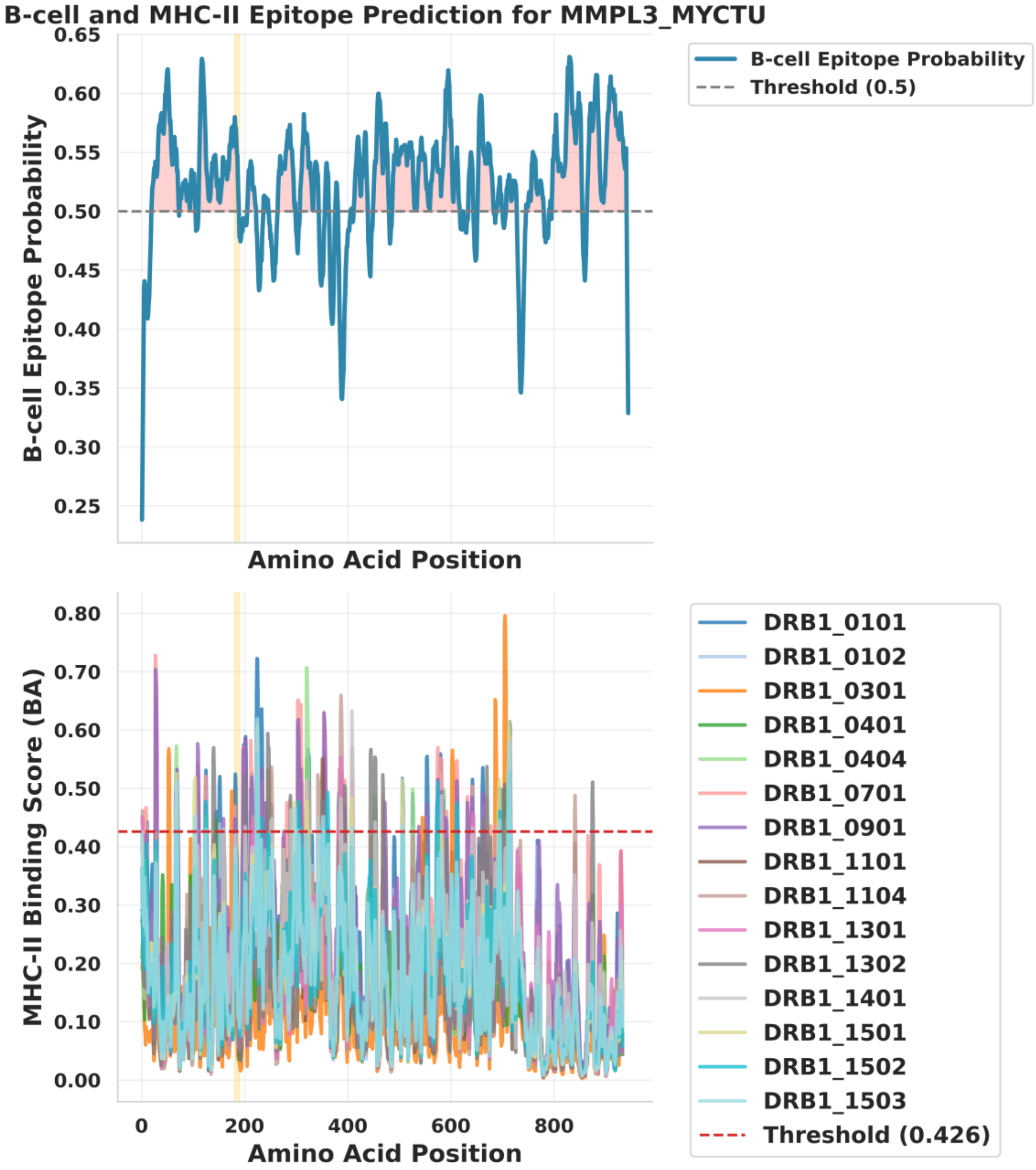

Figure S6. Epitope prediction profile for protein Mmpl3. B-cell (upper) and MHC-II (lower) epitope prediction scores are plotted along the full protein sequence. The dashed line indicates the positive threshold. The candidate peptide region is highlighted in yellow.

Fig. S7

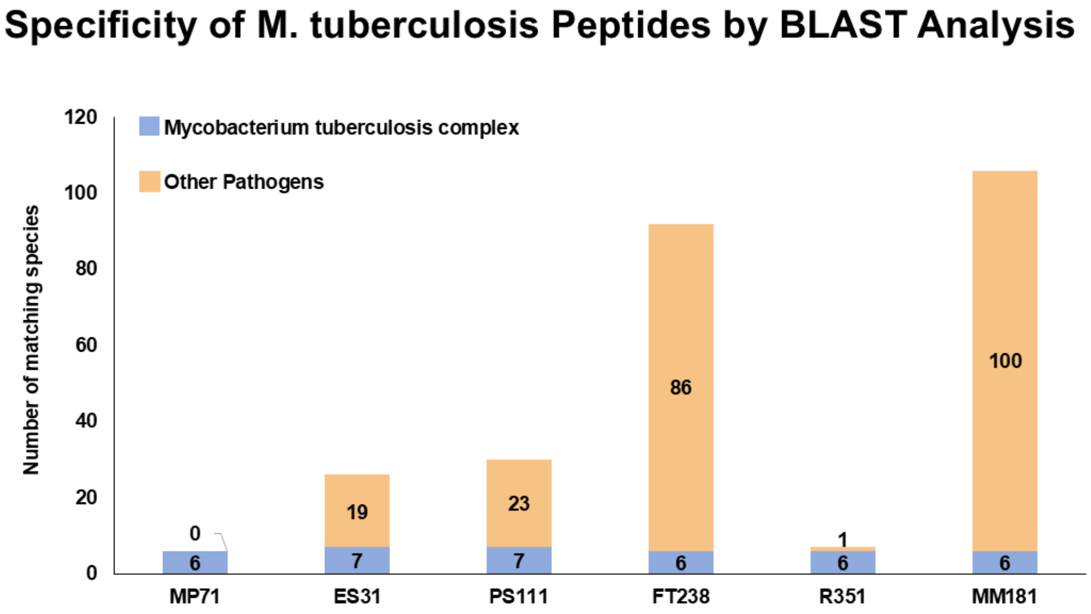

Figure S7. Specificity assessment of candidate peptides. BLAST analysis shows sequence matches for each peptide to the M. tuberculosis complex (lower bar segment) versus other pathogens (upper segment). While some peptides (e.g., MM181, FT238) had broader distribution within the Mycobacterium genus, none showed detectable homology to proteins from common pathogenic species in monkeys, confirming their high specificity for target-based assays.

Fig. S8

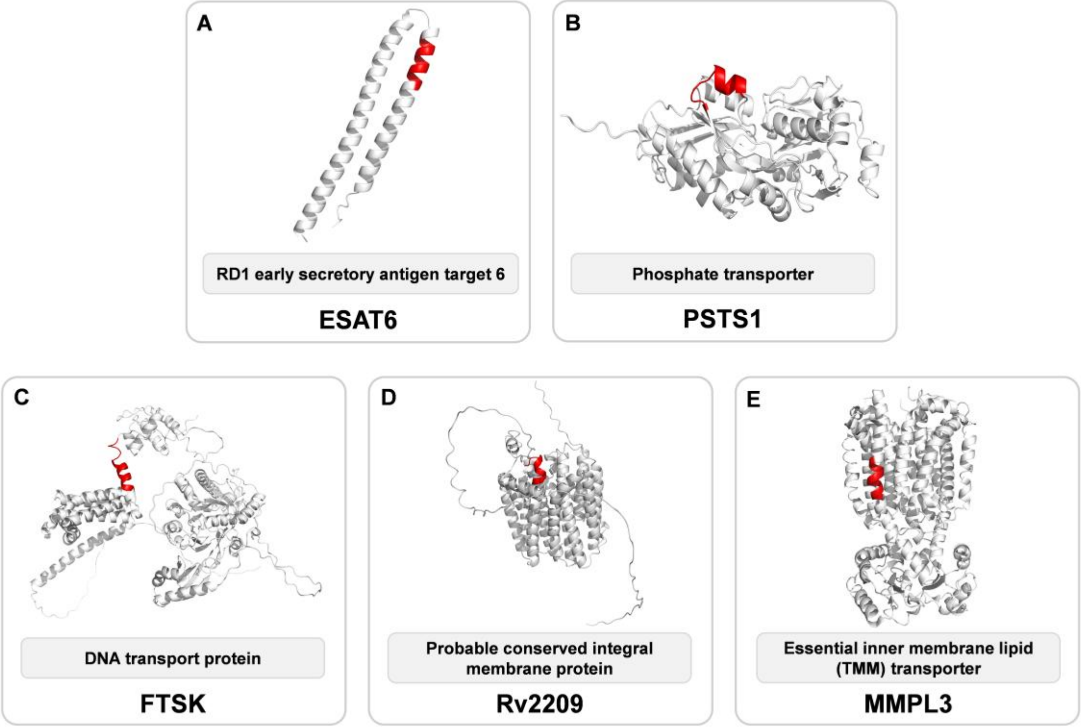

Figure S8. Three-dimensional structures of representative target proteins generated by AlphaFold3 and obtained from RCSB PDB databases, with candidate epitope peptides highlighted in red.

Fig. S9

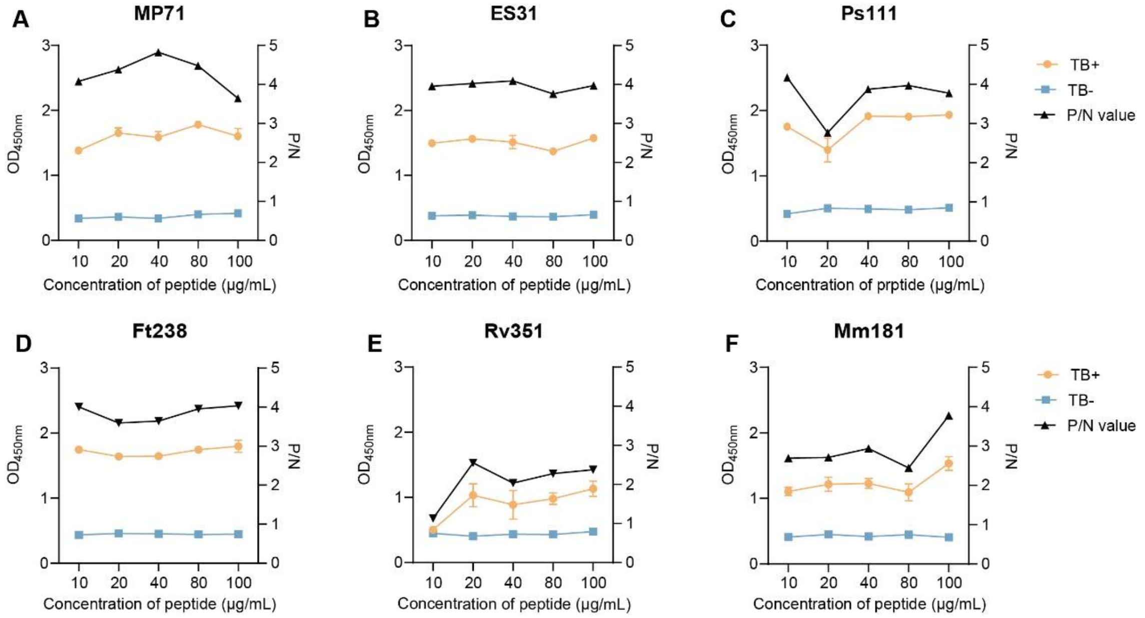

Figure S9. Screening of candidate peptides and assay concentrations. **(A-F)** MP71, ES31, Ps111, Ft238, Rv351, and Mm181 were screened under identical assay conditions: a serum dilution of 1:100, an IgM-HRP secondary antibody dilution of 1:6000, and blocking with 5% skim milk.

Fig. S10

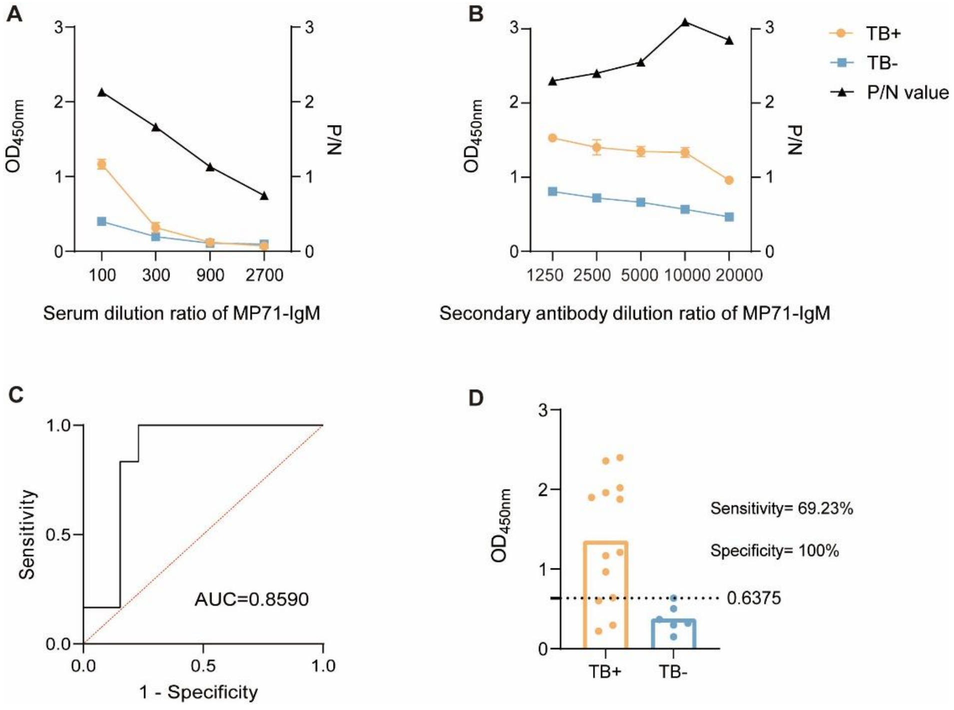

Figure S10. Optimization of assay conditions and Initial AUC assessment. **(A)** Serum dilution factor.The secondary antibody dilution factor was 1:6000. **(B)** Secondary antibody dilution factor (IgM-HRP). The serum dilution factor was 1:100. Both A and B use coating concentrations MP71 (40 µg/mL)and the blocking buffer was 5% skimmed milk. **(C, D)** Evaluation of diagnostic performance

Fig. S11

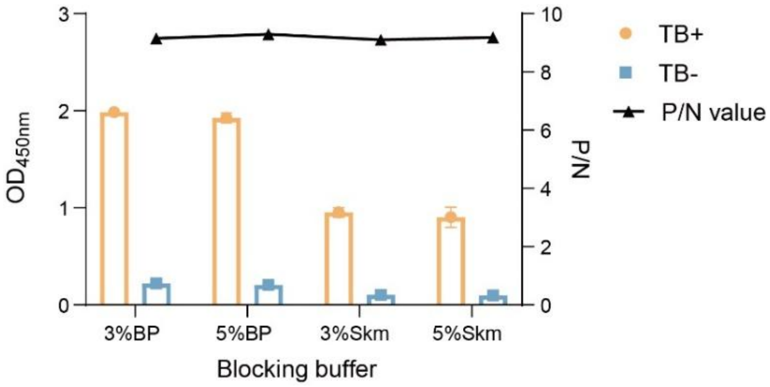

Figure S11. Optimization of experimental conditions. BP means BSA-PBST, Skm means skim milk.
